# Quantum-to-classical crossover near quantum critical point

**DOI:** 10.1038/srep18600

**Published:** 2015-12-21

**Authors:** M. Vasin, V. Ryzhov, V. M. Vinokur

**Affiliations:** 1 Physical-Technical Institute, Ural Branch of Russian Academy of Sciences, 426000 Izhevsk, Russia; 2 High Pressure Physics Institute, Russian Academy of Sciences, Moscow, Russia; 3 Materials Science Division, Argonne National Laboratory, 9700 S. Cass Avenue, Argonne, Illinois 60637, USA

## Abstract

A quantum phase transition (QPT) is an inherently dynamic phenomenon. However, while non-dissipative quantum dynamics is described in detail, the question, that is not thoroughly understood is how the omnipresent *dissipative* processes enter the critical dynamics near a quantum critical point (QCP). Here we report a general approach enabling inclusion of both adiabatic and dissipative processes into the critical dynamics on the same footing. We reveal three distinct critical modes, the adiabatic quantum mode (AQM), the dissipative classical mode [classical critical dynamics mode (CCDM)], and the dissipative quantum critical mode (DQCM). We find that as a result of the transition from the regime dominated by thermal fluctuations to that governed by the quantum ones, the system acquires effective dimension *d* + *z*Λ(*T*), where *z* is the dynamical exponent, and temperature-depending parameter Λ(*T*) ∈ [0, 1] decreases with the temperature such that Λ(*T* = 0) = 1 and Λ(*T* → ∞) = 0. Our findings lead to a unified picture of quantum critical phenomena including both dissipation- and dissipationless quantum dynamic effects and offer a quantitative description of the quantum-to-classical crossover.

A quantum phase transition (QPT), an abrupt change in the ground state of a system due to quantum fluctuations, is driven by a varying controlling parameter at zero temperature *T* = 0, unlike the classical phase transition that can be accessed by temperature^1^. A key component of a quantitative description of quantum critical behavior is mapping the *d*-dimensional quantum system onto a (*d* + 1)-dimensional classical statistical mechanics allowing for the powerful finite size scaling near the quantum critical point (QCP)^1^,^2^. The extra dimension, imaginary time, reflects that the quantum description is inherently dynamic. A quantum second order transition occurs as the controlling parameter goes across the QCP where the quantum fluctuations driving the transition diverge and become scale invariant in space and time. The insights into QPT promise to lead to an understanding of the phase transformations of many body strongly correlated systems, most notably of the mechanisms of high temperature superconductivity in cuprates, which is encoded in the physics of the critical region near QCP. The scaling description of this region rests on the mapping of the *d*-dimensional quantum system to a (*d* + 1)-dimensional classical one^2^,^3^,^4^,^5^,^6^,^7^,^8^,^9^,^10^, where the extra dimension is imaginary time. The immediate corollary of the equivalence between a quantum system and a classical system with an extra “temporal” dimension is along with the usual spatial correlation length diverging on approach to transition as *ξ* ~ |Δ|^−*ν*^, where Δ is the deviation of the controlling parameter from its critical value, there appears a distinct correlation “length,” *ξ*_τ_ ~ *ξ*^*z*^, in the time direction, where *ν* and *z* are the correlation length exponent and the dynamical-scaling exponent, respectively. Temperature introduces a new energy scale, *k*_*B*_*T*/*ħ* separating classical and quantum regimes. Modes with frequencies *ħω* > *k*_*B*_*T* describe quantum fluctuations, while those with *ħω* < *k*_*B*_*T* behave classically^1^,^2^. To complete classification of energies one has to take into account dissipation processes that can drastically change the critical behavior^11^,^12^,^13^,^14^. However, in spite of the extensive attention to and the remarkable progress in description of the dissipative dynamics near a critical point, the thorough understanding of the physics of the dissipative mode of quantum fluctuations is still lacking and is the subject of the intense debate^4^,^5^,^6^,^9^,^15^,^16^,^17^.

Here we take up on this task and develop a unified approach based on the Keldysh description of the of out-of-equilibrium dynamics description, enabling an analytical treatment of the crossover among all three critical modes. We will show, that this crossover does not change the system universality class, and that the modification of the critical exponents is the result of the change of the effective dimensionality of the dynamic system, which accompanies the crossover from CCDM where the thermal fluctuations dominate to DQCM where the quantum fluctuations determine the critical behavior.

Let us consider for concreteness a Bose system described by the one-component order parameter scalar field *ϕ* and with the potential energy given by the functional *U*{*ϕ*}, e.g. *U* ∝ *ϕ*^4^. We take the units in which *ħ* = 1 and *k*_*B*_ = 1. The partition function of the system is given by


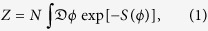


where 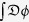 denotes the functional *ϕ*-field integration and the action, *S*, is defined as


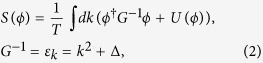


where *U* = *vϕ*^4^, Δ is the governing parameter, that tends to zero at the critical point. To describe quantum critical dynamics we employ the Keldysh technique, initially formulated for quantum systems, where the role of the partition function is played by the functional path integral which after the Wick rotation assumes the form^18^:


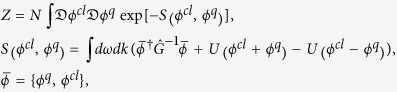


where *ϕ*^*q*^ and *ϕ*^*cl*^ are pair of fields called “quantum” and “classical” respectively, the matrix of the inverse correlation functions is^18^


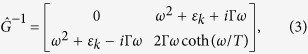


Γ is the kinetic coefficient, and the function coth(*ω*/*T*) is the function of the density of states of the ideal Bose gas. The advanced, retarded, and Keldysh parts of both the correlation functions matrix and the inverse matrix are connected by the fluctuation-dissipation theorem (FDT): 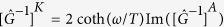. These expressions set the ground for quantitative description of critical dynamics in the vicinity of the critical point. They are general and allow the system to be described both within the classical, *T*  ≫ *ω*, and the quantum, *T* ≪ *ω*, limits.

Let us start with the region of the dominance of thermal fluctuations, *ω* ≪ *T*, which includes, in particular, the plane *ω* = 0. The critical dynamics of the system is determined by the Keldysh element of the Green function matrix, 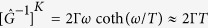. Note that in this region the influence of the thermostat on the system (i.e. the action of the statistical ensemble on its own element) corresponds to the action of the external “white” noise. The low energy long wave fluctuations, *k* → 0, *ω* → 0, are relevant near the critical point, therefore only the terms with the lowest powers *k* and *ω* are to be kept. Hence in the fluctuation region the system is described by the classical non-equilibrium propagator:


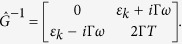


satisfying the standard FDT:


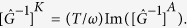


Accordingly, the dispersion relation is *ω* ∝ *k*^2^, therefore the dynamic critical exponent (the scaling dimension) in the first approximation is *z* = 2. Now, as usual, going over from statics to the dynamic description implies using the total (space + time) dimensionality *D* = *d* + *z*, where the spatial dimensionality, *d*, controlling the static critical behavior (in the dissipative systems with the non-conserving order parameter is usually also *z* = 2), so that *D* = *d* + 2. However the ‘white noise’ reduces the effective scaling dimensionality to *D*_*eff*_ = *D* − 2 = *d*^19^. As a result, the critical dimensions of the dynamic and static theories coincide, and the critical behavior of the system is described by the classical critical dynamics of the *d*-dimensional system. We will be referring hereafter to this mode as to the classical critical dynamics (CCDM), that realizes at *T* ≫ *ω*, Γ*T* ≫ |Δ|.

Now let us turn to the quantum fluctuations domain, *ω* > *T*, and consider the critical fluctuation regime, Γ*ω* > Δ, so that we can not neglect the dissipation processes. Similarly to the above, we can neglect the term with *ω*^2^ in the Green function, since *ω* → 0. Then the inverse correlation function defined by Equation (3) reduces to:


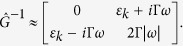


In the quantum limit the FDT assumes the form


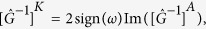


and the action of the statistic ensemble on the system does not depend on the temperature. Note, that close to the phase transition (i.e. at Δ ≈ 0), where *ε*_*k*_ → 0, the Keldysh Green function becomes *G*^*K*^(*ω*) = 2/(Γ|*ω*|), and describes the 1/*f*-noise with the intensity independent of the temperature. This significantly changes the critical properties of the system as compared to those in *ω* ≪ *T* case. The total dimension now remains *D* = *d* + 2. However, the 1/*f* noise in contrast to the “white”-noise, does not decrease the effective scaling dimension^20^, therefore the effective dimension of the dissipative quantum system is greater by 2 than its static dimension, so that *D*_*eff*_ = *d* + 2. The disagreement between the static and dynamic theories is accounted for by the fact that in the quantum case there is no static limit, and the only correct results are those of the dynamic theory. The corresponding dynamic mode can be referred to as the dissipative quantum critical mode (DQCM), *T* ≪ *ω*, Γ*ω* ≫ |Δ|.

If the quantum limit, *ω* > *T*, still holds, but the coherence time becomes shorter than the inverse frequency of quantum fluctuations, Γ*ω* ≪ |Δ|, the system dynamics is governed by the adiabatic mode, in which the dissipation can be neglected. Therefore we let Γ = 0, and from Equation (3) find


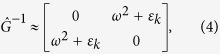


with the dispersion relation becoming *ω* ∝ *k*. Accordingly, the critical dynamic exponent is *z* = 1, and the critical behavior is that of the static system with the effective dimensionality *D*_*eff*_ = *d* + 1. Furthermore, the critical behavior of the three-dimensional system is described, in this parameters range, by the mean field Ginzburg–Landau theory, since the effective dimensionality is equal to the critical one, 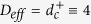. This regime can be referred to as the adiabatic quantum mechanical mode (AQM), *T* ≪ *ω*, Γ*ω* ≪ |Δ|.

Our classification of different critical regions is summarized by the phase diagram shown in Fig. 1. The surfaces mark crossovers between different critical regimes corresponding to different critical modes. The plane *ω* = *T* separates the quantum- and thermal fluctuations-dominated regions. This crossover can be observed, for example, by the change in the exponent *β*, which at *T*/*ω* ≪ 1, assumes the value corresponding to the critical exponent of the mean field theory, since the effective dimension of the system becomes greater than the critical dimension, 
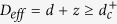
, whereas at *T*/*ω* ≫ 1, *β* is equal to the value characteristic to the three-dimensional classical system. This behavior contrasts to what one would have expected from the orthodox viewpoint since the universality class that determines the value of the exponent is not supposed to vary with the temperature. The reason is that the value of the critical exponent depends also on the nature of the critical fluctuations. Accordingly, the change in *β* reflects the increase in the effective dimensionality accompanying the crossover from the thermal fluctuations- to the quantum fluctuations mode, the system’s universality class remaining intact. It can be shown that the crossover occurs due to the changes in the system density of states when the temperature approaches to zero. To do so one notices that all graphs giving major contribution to the renormalization, contain the loop consisting only of one advanced (or retarded) Green function and one Keldysh Green function^20^ [see Supplementary Information (SI)]. In the long-wavelength limit corresponding to the critical dynamics near the phase transition, the contribution of this loop is


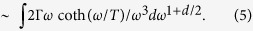


In order to reduce the renormalization procedure to the standard form one should approximate function *ω* coth(*ω*/*T*) by an exponential function, 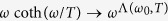 near the low-frequency cut off *ω* = *ω*_0_. Then the renormalization procedure becomes the standard problem of describing the critical behavior of the *d* + 2Λ-dimensional system, where Λ is the function of the temperature: Λ(*ω*_0_/*T*) = (coth(*ω*_0_/*T*) − (*ω*_0_/*T*)csch^2^(*ω*_0_/*T*))tanh(*ω*_0_/*T*) (see SI).

Using this approximation one can estimate the temperature dependence of the critical exponents, which characterize the heat capacity, *C*_*v*_ ~ |Δ|^−*α*^, susceptibility, *χ* ~ |Δ|^−*γ*^, magnetization, 〈*φ*〉 ~ |Δ|^*β*^, correlation radius, *r*_*c*_ ~ |Δ|^−*ν*^, and Green function, *G*(*r*) ~ *r*^−*d*+2−*η*^ (*η* is the anomalous dimension index). In the case of the crossover from CCDM to DQCM, where *d* → *d*′ = *D*_*eff*_ = *d* + 2Λ, the standard relations for the critical exponents also hold for the effective exponents, *ν* → *ν*′(Λ), *η* → *η*′(Λ), *β* → *β*′(Λ), *γ* → *γ*′(Λ), *α* → *α*′(Λ). The latter can be calculated from the well known expressions obtained in the framework of the *ε*-expansion for the 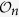-*φ*^4^-model^21^,^22^,^23^: 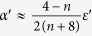, 
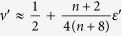
, 
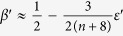
 and 
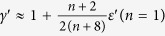
, where *ε*′ = (4 − *d*′)/2 = (4 − *d*)/2 − Λ = *ε* − Λ. Using these formulas and the dependence Λ(*ω*_0_/*T*) we calculate functions *β*′(*T*), *ν*′(*T*), *γ*′(*T*), and *α*′(*T*), see Fig. 2. One sees that *β*′(*T*) dependence is in a good qualitative agreement with the experimental data^24^.

The exponent Λ is equal to 1/2 at *T*/*ω*_0_ = 1, as shown in Fig. 1. In this case the effective dimensionality is equal to the upper critical dimensionality of the system, *D*_*eff*_ = 4, and all the critical exponents reach their mean-field values. They do not vary at lower temperatures in the mean-field region, where the residual quantum fluctuations already cannot destroy the order. In the two-dimensional system the critical exponents assume their mean-field values at *T*/*ω*_0_ = 0. In the one-dimensional system the quantum fluctuations also cannot destroy the order, and the critical exponents are calculated from the standard scaling theory.

Our results show that the critical behavior in the vicinity of the quantum critical point is multi-critical. The functional technique of theoretical description of non-equilibrium dynamics allows us to describe the entire spectrum of critical modes in the vicinity of quantum phase transition within a single formalism. In particular, it describes the crossover between CCDM and DQCM, and the unusual temperature dependence of the system critical exponents. Note that in this case the system universality class does not change. The continuous change of the critical exponents is the dynamical effect, which is caused by the crossover from the thermal fluctuation mode to the quantum fluctuation mode. The crossover between these critical modes was experimentally observed in^24^,^25^,^26^. The results of^26^ favors the quantum concept of effective increasing space dimensionality at low temperatures that suppresses a fluctuation divergence at a second order phase transition, in^24^ it is shown that the crossover from the classical to the quantum criticality takes place with the corresponding continuous change of the critical indexes, see Fig. 2. One can see that *β*′(*T*) dependence is in a good qualitative agreement with the experimental data^24^, see Fig. 3.

One of the main consequences of our work is that due to the presence of dissipation, the experimental values of the critical exponents of one- and two-dimensional systems close to QCP can differ from the values obtained by the standard rule according to which they would have coincided with the exponents of a (*d* + 1)-dimensional system. We have shown that the critical exponents of a two-dimensional dissipative system close to QCP coincide with the exponents predicted by the mean-field theory, while in a one-dimensional system the exponents coincide with those of the three-dimensional classical system. The obtained results well describe the available experimental data^24^,^25^,^26^. Our findings explain the unexpected temperature dependence of the critical exponents in the vicinity of quantum critical points and open the route for further experimental investigation of the quantum-to-classical crossover in the vicinity of the quantum critical point.

## Methods

We consider the quantum critical dynamics of the Ginsburg-Landau model in terms of the Keldysh technique^18^. The Lagrangian of this model has the following form:


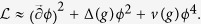


where *ϕ* is the scalar order parameter field, which obeys to the Bose statistics. We suppose Δ and *v* to depend on some external parameter *g*, that controls the system state.

In the Keldysh technique the description of the non-equilibrium dynamics of the system is performed using the generating functional, which is written in the form of


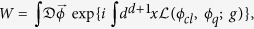


where 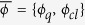, *ϕ*_*cl*_ and *ϕ*_*q*_ are the “classical” and “quantum” parts of the order parameter accordingly, and 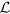 is the fields lagrangian density. For Bose system in the frequency representation it has the form of:


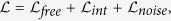


where


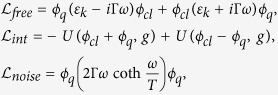


*ε*_*k*_ = *k*^2^ + Δ(*g*), Γ is the kinetic coefficient, and *U*(*ϕ*) = *v*(*g*)*ϕ*^4^ is the interaction part. 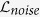 is Keldysh part of the lagrangian density of the ideal Bose gas. We consider the crossover of the critical dynamics of the system, caused by the continuous change of temperature from the high values, *T* → ∞, to the zero limit, *T* → 0 (see SI).

The analysis of the critical dynamics is carried out with help of the renormalization group procedure presupposing the fairness of the dynamical scaling hypothesis. In terms of this approach the canonical dimensions (the effective dimensionality values, which are included in the calculation of the critical exponents in the dynamical case) of the fields and the model parameters are determined from the condition of dimensionless action. The corresponding summarized canonical dimensions, *D*[*F*], of any values, *F*, are determined as:


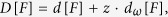


where *d*_*ω*_[*F*] is the frequency dimension^1^,^23^. We carry out the standard procedure of the critical exponents calculation using the renormalization with the momentum cutoff, *ω*_0_ → *λω*_0_ (*λ* is the cutoff parameter). In the one-loop approximation the renormalization group of the model under study has the form:


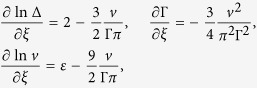


where *ξ* = ln(1/*λ*), *ε* = 4 − *z*Λ − *d*, and Λ is the temperature-dependent parameter, which eventually defines the temperature dependence of the critical exponents (see SI).

## Additional Information

**How to cite this article**: Vasin, M. *et al.* Quantum-to-classical crossover near quantum critical point. *Sci. Rep.* **5**, 18600; doi: 10.1038/srep18600 (2015).

## Supplementary Material

Supplementary Information

## Figures and Tables

**Figure 1:**
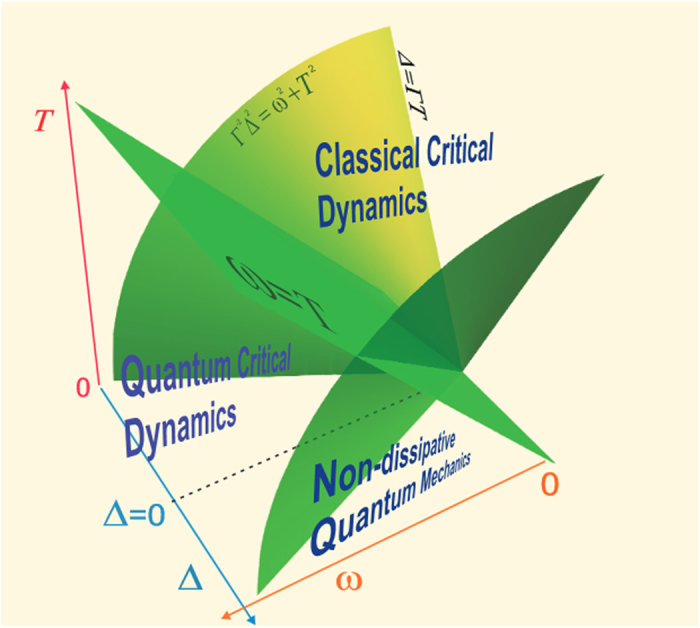
Phase diagram displaying different fluctuation regimes. The green color denotes the conventional surface *ω*^2^ + *T*^2^ = |Δ|^2*ν*^ showing the location of the crossover region between the dissipative and adiabatic fluctuation modes.

**Figure 2:**
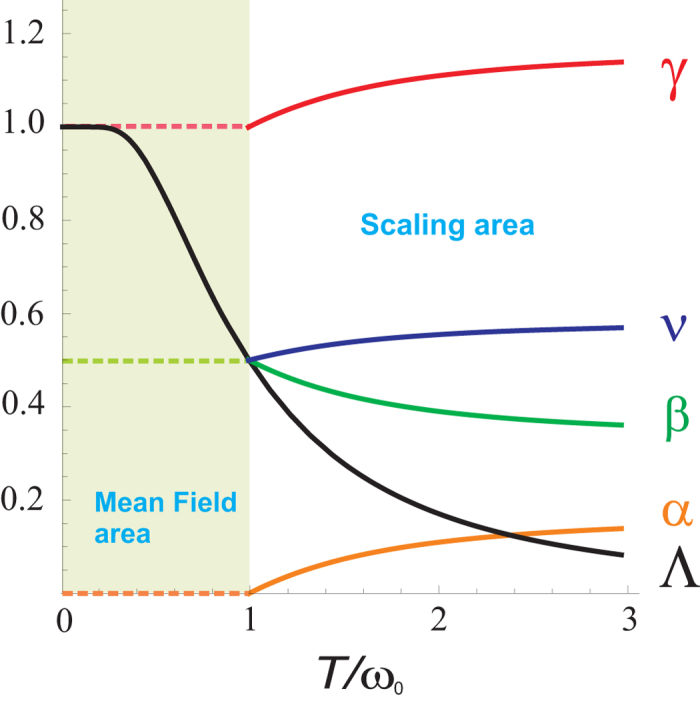
Critical exponents. Theoretical dependence of Λ (pink line), and the critical exponents: *β*′ (green line), *α*′ (black line), *ν*′ (red line), and *γ*′ (blue line) on the *T*/*ω*_0_ ratio for the three-dimensional *ϕ*^4^-model. When Λ becomes to be equal to 1/2 and, accordingly, *D*_*eff*_ = 4, then all exponents take the mean-field values.

**Figure 3:**
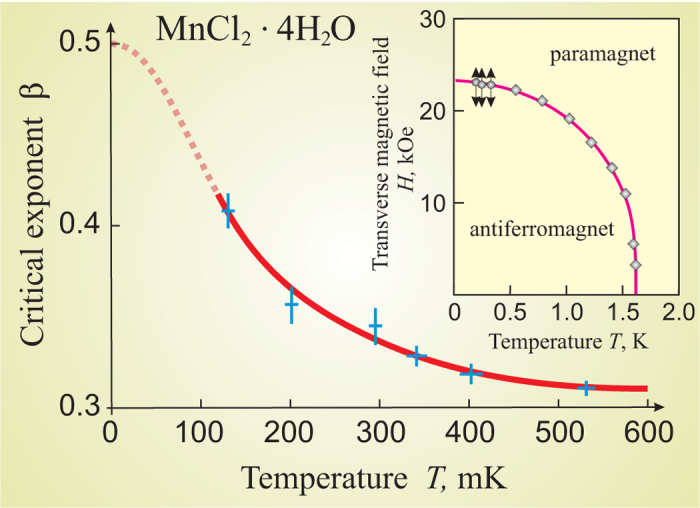
Experimental data. Dependence of the critical exponent *β* determining the order parameter behavior of the antiferromagnet MnCl_2_ ⋅ 4H_2_O on the temperature from refs 24.
