# Supplementary material for: Quantum-to-classical crossover near quantum critical point
Source: Sci Rep. 2015 Dec 21;5:18600. doi: 10.1038/srep18600 (PMC4685645; doi:10.1038/srep18600)
Supplement: Supplementary Information [file srep18600-s1.pdf]

Supplementary Information: Quantum-to-classical crossover near quantum critical point

M. Vasin,[1,2] V. Ryzhov,[2] & V. M. Vinokur[3]

August 1, 2015

1 Quantum critical dynamics in the Keldysh technique

Dynamics the quantum critical in the framework of the Ginsburg-Landau model is described in terms of the Keldysh technique. The Lagrangian has the form:

$$\mathcal{L}\approx(\vec{\partial}\phi)^2+\Delta(g)\phi^2+v(g)\phi^4\,,$$

where ϕ is the scalar order parameter field, which obeys to the Bose statistics. We suppose Δ and v to depend on some external parameter g, that controls the system state. The system interacts with a heat bath having the temperature T. The generating functional for non-equilibrium system has the form

$$W=\int\mathfrak{D}\vec{\phi}\exp\left\{i\int d^{d+1}x\mathcal{L}(\phi_{cl},\,\phi_q;\,g)\right\},$$

where $\vec{\phi}=\{\phi_q,\,\phi_{cl}\}$, ϕ_{cl} and ϕ_q are the "classical" and "quantum" parts of the order parameter accordingly, and $\mathcal{L}$ is the fields Lagrangian density. Using the Wick rotation, $t=-ix_4$, we move from the Minkowski space to the Euclidean one. Then

$$W=\int\mathfrak{D}\vec{\phi}\exp\left\{-\int d^dkd\omega\mathcal{L}(\phi_{cl},\,\phi_q;\,g)\right\}\,.$$

The coupling of the system with an arbitrary environment, including an external noise, is described as the interaction with the 'effective' heat bath, whereas the 'internal (quantum) noise' is supposed to be built in directly into the model. Then according to [S1] the Keldysh Lagrangian assumes the form

$$\mathcal{L}=\mathcal{L}_{free}+\mathcal{L}_{int}+\mathcal{L}_{noise},$$

where

$$\mathcal{L}_{free}=\phi_q\,(\varepsilon_k-i\Gamma\omega)\,\phi_{cl}+\phi_{cl}\,(\varepsilon_k+i\Gamma\omega)\,\phi_q,$$

$$\mathcal{L}_{int}=-\,U(\phi_{cl}+\phi_q,\,g_{cl}+g_q)+U(\phi_{cl}-\phi_q,\,g),$$

$$\mathcal{L}_{noise}=\phi_q\left(2\Gamma\omega\coth\frac{\omega}{T}\right)\phi_q,$$

$\varepsilon_k=k^2+\Delta(g)$, and $U(\phi)$ is the interaction energy. The expressions for Retarded, Advanced and the Keldysh components of the matrix Green function are:

$$G^K=G^R\circ F-F\circ G^A,$$

where F is the Hermitian matrix ($F=F^{\dagger}$), and the circle stands for the integration over the intermediate time (matrix multiplication) [S1]. One can check that

$$[G^{-1}]^K=[G^R]^{-1}\circ F-F\circ[G^A]^{-1}.$$

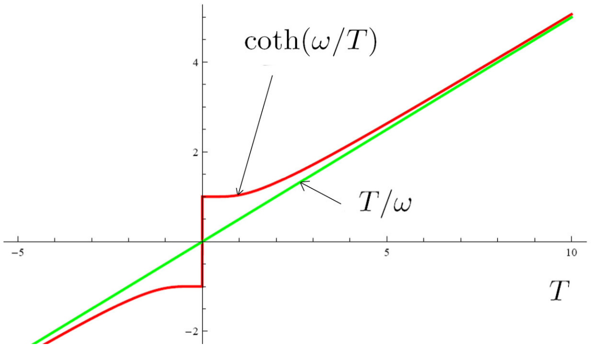

Figure S 1 | Temperature dependence of $\coth(\omega/T)$**.** The red line depicts $\coth(\omega/T)$ versus T function (with $\omega=4$), the green line is the T/ω function. At high temperatures these graphics coincide, which corresponds to the critical dynamics. However, $\coth(\omega/T)\to\mathrm{sign}(\omega)$ close to $T=0$, where the system is described by the quantum critical dynamics.

After the Wigner transform (WT) in the frequency representation we arrive at

$$G^K=f(\omega)(G^R-G^A),$$

$$[G^{-1}]^K=f(\omega)\left([G^R]^{-1}-[G^A]^{-1}\right),$$

where $f(\omega)$ is the distribution function. For a boson system in thermal equilibrium $f=-i\coth(\omega/T)$, where T is the temperature of the heat bath [S1]. This expresses the fluctuation-dissipation theorem (FDT).

We consider the system with the dissipation; then

$$[G^R]^{-1}=\varepsilon_k+i\Gamma\omega,\quad [G^A]^{-1}=\varepsilon_k-i\Gamma\omega,$$

$$[G^{-1}]^K=2\Gamma\omega\coth(\omega/T),$$

where Γ is the kinetic coefficient. In the quantum case $T\ll\omega$ (see Fig. 1)

$$\coth(\omega/T)\to\mathrm{sign}(\omega)\quad\Rightarrow\quad[G^{-1}]^K=2\Gamma|\omega|.$$

The FDT has the following form: $G^K=i\,\mathrm{sign}(\omega)(G^R-G^A)$. In the classical case $T\gg\omega$ (see Fig. 1)

$$\coth(\alpha\omega)\to\frac{T}{\omega}\quad\Rightarrow\quad[G^{-1}]^K=2\Gamma T,$$

and the system satisfies the usual classical form of FDT: $G^K=T(G^R-G^A)/i\omega$.

Below we will concentrate on the quantum limit ($\omega\gg T\approx0$), when $\coth(\omega/T)\to\mathrm{sign}(\omega)$, the temperature is not essential in the FDT,

[1]Physical-Technical Institute, Ural Branch of Russian Academy of Sciences, 426000 Izhevsk, Russia; [2]High Pressure Physics Institute, Russian Academy of Sciences, Moscow, Russia [3]Materials Science Division, Argonne National Laboratory, 9700 S. Cass Ave, Argonne, IL 60439, USA

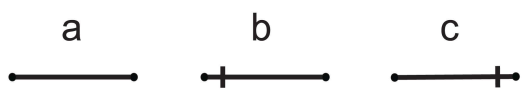

Figure S 2 | The diagrammatic representation of the components of the matrix Green function. **a**: Keldysh Green function, G^K; **b**; advanced Green function, G^A; **c**: retarded Green function, G^R.

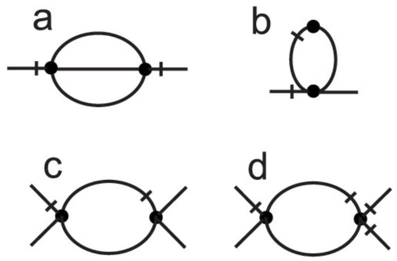

Figure S 3 | The diagrammatic representation of the contributions to the renormalization of the vertices. **a**: Renormalization of Γ, **b**: Renormalization of Δ, **c** and **d**: renormalization of v.

$\mathcal{L}_{noise} = \phi_q\,(2\Gamma|\omega|)\,\phi_q$, and the Keldysh Green function has the following form:

$$G^K(\omega) = \frac{2\Gamma|\omega|}{\varepsilon_k^2 + \Gamma^2\omega^2}.$$

Note, that close to the phase transition ($\Delta \approx 0$), when $\varepsilon_k \to 0$, we get $G^K(\omega) = 2/\Gamma|\omega|$. This is the so called $1/f$-noise, whose intensity does not depend on the temperature but it is equal to $\hbar$. One can infer that the presence of $1/f$-noise is a natural property of the cold many-body Bose system, which follows from the quantum character of dynamics in $T = 0$. This justifies Eq. (4) of the main text.

2 Quantum critical dynamics of the d-dimensional Ginsburg–Landau model

We suppose that the system is close to the second order phase transition, when the interaction part of the action can be written as

$$U \approx \Delta(g)\phi^2 + v(g_c)\phi^4,$$

where $\Delta(g) = |g - g_c| \to 0$ close to the phase transition point, g_c.

Below we will consider the critical dynamics of this system in the d-dimensional space near the critical point. The critical dynamics rests on the hypothesis of dynamic scaling, according to which the action should be invariant with respect to the scale transformations relating frequency and the wave vector as $\omega \propto k^z$, with z being the so-called dynamic critical exponent. The effective dimension, $D = d + z$, takes the role of the conventional (momentum) dimension, d_k, in the static case.

The canonical dimensions of the fields and the model parameters are determined from the condition that the action is dimensionless. The corresponding effective canonical dimensions, $D[F]$, of a variable F, are defined as:

$$D[F] = d[F] + z \cdot d_\omega[F],$$

where $d_\omega[F]$ is the frequency dimension [S2, S3]. The canonical dimensions of the values of our theory are given in the table:

F	k	ω	$\phi_{\rm cl}$	$\phi_{\rm q}$	v	Γ	Δ
$d[F]$	1	0	$\frac{d}{2}-1$	$\frac{d}{2}+1$	$4-d$	2	2
$d_\omega[F]$	0	1	$-\frac{\Lambda}{2}$	$-\frac{\Lambda}{2}$	$-\Lambda$	-1	0
$D[F]$	1	2	$\frac{d}{2}-\Lambda-1$	$\frac{d}{2}-\Lambda+1$	$4-2\Lambda-d$	0	2

The renormalization procedure is carried out by the standard method. It is assumed that the fields $\phi_{\rm q}$, and $\phi_{\rm cl}$ are the slow-varying ones, so that the Fourier-transformed fields have only long-wave components: $|k| < k_0$; $\omega < \omega_0$. At the first step of RG transformations one integrates the partition function over the components of the fields in the limited wave band $\lambda k_0 < k < k_0$, $\lambda^z\omega_0 < \omega < \omega_0$. The renormalized parameters have the following form:

$$\begin{aligned}\Delta^{(R)} &= Z_\Delta Z_{\phi_{\rm q}} Z_{\phi_{\rm cl}} \lambda^{d+\varepsilon+z} = Z_\Delta \lambda^{-2},\\ \Gamma^{(R)} &= Z_\gamma Z_{\phi_{\rm q}}^2 \lambda^{d+\varepsilon+2z} = Z_\Gamma \lambda^0,\\ v^{(R)} &= Z_v Z_{\phi_{\rm q}} Z_{\phi_{\rm cl}}^3 \lambda^{3d+3\varepsilon+3z} = Z_v \lambda^\varepsilon\,,\end{aligned}$$

where $Z_{\phi_{\rm q}}, Z_{\phi_{\rm cl}}, Z_\Delta, Z_v, Z_\Gamma$ are the constants of renormalization, and $\varepsilon = 4 - z\Lambda - d$ is the small parameter of the ε-expansion.

To gain an insight into the renormalization procedure let us follow the renormalization of Δ as an example in detail. We will limit ourselves to one loop approximation which is sufficient to capture all the distinctive features of the theory. The main divergent contributions in the diagrammatic representation is shown in Fig. 3b, and the renormalization of Δ has the form:

$$Z_\Delta \simeq \Delta - \frac{6\Delta v}{(2\pi)^3}\int\limits_{\lambda^z\omega_0}^{\omega_0}\int\limits_{\lambda k_0}^{k_0} G^K(k,\omega)G^R(k,\omega)d\mathbf{k}d\omega =$$

$$= \Delta - \frac{12\Delta v\pi^2}{\Gamma(2\pi)^3}\int\limits_{\lambda k_0}^{k_0}\frac{dk}{k} = \Delta - \frac{3\Delta v}{2\Gamma\pi}\ln(1/\lambda).$$

One sees that the integral in this expression diverges logarithmically if the momentum dimension is $d[k] \equiv d = 4 - z\Lambda$. Then renormalized Δ is

$$\Delta^{(R)} = e^{2\xi}Z_\Delta \simeq e^{2\xi}\left[\Delta - \frac{3}{2}\frac{\Delta v}{\Gamma\pi}\,\xi\right],$$

where $\xi = \ln(1/\lambda)$ is the logarithmically divergent factor. In the same way one can get other terms of the renormalized action:

$$v^{(R)} = e^{\varepsilon\xi}Z_v \simeq e^{\varepsilon\xi}\left[v - \frac{9}{2}\frac{v^2}{\Gamma\pi}\,\xi\right].$$

The contribution to the renormalization of the kinetic coefficient $|\omega|\Gamma$ is proportional to $|\omega|$:

$$\Gamma^{(R)} = \Gamma - \frac{3v^2 16\pi^4}{(2\pi)^6\gamma^2}\ln(1/\lambda) = \Gamma - \frac{3v^2}{4\pi^2\Gamma^2}\,\xi.$$

Hence, in the one-loop approximation the renormalization group equations assume the form:

$$\frac{\partial\ln\Delta}{\partial\xi} = 2 - \frac{3}{2}\frac{v}{\Gamma\pi}, \qquad \frac{\partial\Gamma}{\partial\xi} = -\frac{3}{4}\frac{v^2}{\pi^2\Gamma^2},$$
$$\frac{\partial\ln v}{\partial\xi} = \varepsilon - \frac{9}{2}\frac{v}{\Gamma\pi}.$$

From the condition of the stable point existence, $\partial\ln(v)/\partial\xi = 0$, we obtain $v = 2\gamma\pi\varepsilon/9$. Thus, the renormalization equations give $\ln\Delta = \ln|g - g_c| \propto \xi$ and $\Gamma \propto -\xi \propto -\ln|g - g_c|$. Note that if $d = 4 - z\Lambda$, then $v = 0$. In this case only the quadratic term is relevant so that the critical behavior is well described by the Gaussian theory.

The approximation of function $f(x) = x\coth(x)$, which enters the Keldysh Green function, is the major problem of this work. The representation of the approximating function as a power function, $f(x) \approx x^{\Lambda(x)}$, enables performing the renormalization group analysis, since the exponent-like behavior of this function defines the condition for the logarithmic divergence of the loop integral. Let us plot $x\coth(x)$ in logarithmic coordinates, see Fig. 4. The plot shows that the slope of the tangent to this graph varies monotonically from 1 for large values of $x \gg 1$ to near zero for small values of $x \ll 1$. Therefore, in the neighborhood of each point x' the function $x\coth(x)$ can be approximated by the function $x^{\Lambda(x')}C(x')$, where $\Lambda(x') = \partial\ln[x\coth(x)]/\partial\ln x|_{x=x'}$ $(0 \leqslant \Lambda \leqslant 1)$. Thus, the exponent Λ can be represented in the follow form: $\Lambda(x') = (\coth(x') - (x')\,\mathrm{csch}^2(x'))\tanh(x')$. This justifies Eq. (7) of the main text.

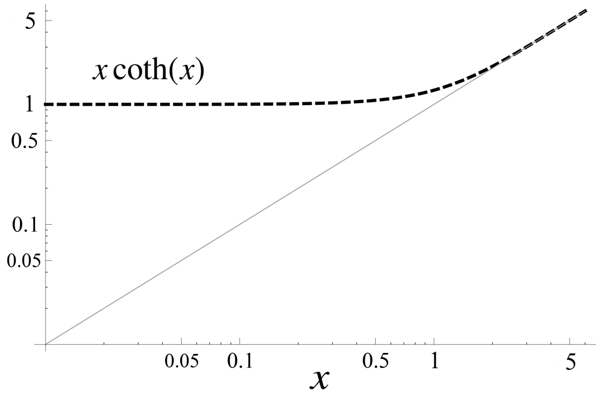

Figure S 4 | The $f(x) = x\coth(x)$ function is shown by the dashed line.

Supplementary References

[S1] Sachdev, S., Quantum Phase Transitions, Second Edition. *CAMBRIDGE UNIVERSITY PRESS* (2011).

[S2] Vasil'ev, A. N., Quantum-Field Renormalization Group in the Theory of Critical Phenomena and Stochastic Dynamics. *CRC Press, Boca Raton, London, New York, Washington* ISBN: 0415310024, (2004);

[S3] Patashinskii, A. Z., Pokrovskii, V. L., Fluctuation Theory of Phase Transitions. *Pergamon Press, Oxford, New York, Toronto, Sydney, Paris, Frankfurt* (1979);
